# Memantine in neurological disorders – schizophrenia and depression

**DOI:** 10.1007/s00109-020-01982-z

**Published:** 2021-01-14

**Authors:** Kamila Czarnecka, Jakub Chuchmacz, Przemysław Wójtowicz, Paweł Szymański

**Affiliations:** Department of Pharmaceutical Chemistry, Drug Analyses and Radiopharmacy, Faculty of Pharmacy, Medical University of Lodz, Muszynskiego 1, 90-151 Lodz, Poland

**Keywords:** Memantine, Alzheimer’s disease, Schizophrenia, Depression

## Abstract

Memantine is used in Alzheimer’s disease treatment as a non-competitive modern-affinity strong voltage-dependent N-methyl-D-aspartate receptor antagonist. The fundamental role of these receptors is to bind glutamate: the main excitatory neurotransmitter in the brain, believed to play a crucial role in neuronal plasticity and learning mechanisms. Glutamate transmission plays an important role in all internal CNS structures and maintains the physiological state of the brain. Excessive glutamate transmission can lead to enlarged calcium ion current which may cause neurotoxicity; however, insufficient transmission can drastically alter the information flow in neurons and the brain, potentially causing schizophrenia-like symptoms by replacing lost information with completely new stimuli. Hence, it is possible that the modulation of NMDA activity may give rise to pathophysiological states. Available literature and clinical trials indicate that memantine is well tolerated by patients, with very few and light side effects. There is a belief that memantine may also benefit other conditions such as schizophrenia and depression.

## Introduction

Memantine is used for treating patients with Alzheimer’s disease (AD), with various degrees of severity. This common neurodegenerative disorder, characterized by progressive loss of memory and cognitive function, is currently incurable and affects around 15 million people worldwide [1]. Initial symptoms usually appear after the age of 65 and become more severe with age, with the prevalence increasing from 0.5% at age 65 to as high as 8% by 85. AD currently accounts for around 50–75% of dementia cases [2]. Dementia itself has been known by humans for centuries and it is major cause of dependence, mortality, and disability [3]. Currently, over 44 million people worldwide are affected by dementia, and this number is expected to triple by the year 2050 [2, 3]. In 2015, dementia contributed to 11.6% of all deaths in England and Wales and the number of people suffering from dementia is still rising [4–7].

Effective treatment is complicated by a rage of sociological issues, such as low community awareness [8], and the lack of valid epidemiological data. However, we can say that every generation is different: both their level of education and, unfortunately, interest in health vary considerably. In addition, levels of medical care and established medical factors such as cardiovascular risk, diabetes, or metabolic syndrome differ between nations, and human life span is continually extending [7, 9]. Recent reports indicate that age-related symptoms of dementia will significantly decrease in people born in the second part of the twentieth century [10]; these may provide a better understanding of potentially modifiable factors of dementia [10, 11]. Significant roles are also played by the socioeconomic status of the patient and the local community: communities in less developed countries are more likely to experience non-communicable diseases (NCDs), and are more exposed to their causative factors, such as smoking, alcohol intake, and health policies [11].

The literature includes no records of dementia until 1907, when Alois Alzheimer reported the case of a woman who experienced memory loss, believed to have arisen as a consequence of her jealousy toward her husband; she also demonstrated abnormal behavior, such as collecting and misplacing items and a belief that someone was spying on her and was trying to kill her [12]. After her death, autopsy indicated the presence of neurofibrillary tangles and senile plaques in the neocortex and hippocampus [12]. These signs are now known as B-amyloid peptide plaques, comprising elements of degenerated neurons, lost neurons, and synapses, and tangles composed of improperly phosphorylated tau-protein [13]. Their presence is believed to reflect the initial pathogenesis of AD. According to the amyloid hypothesis [3], AD progresses together with the accumulation of a pathological peptide (B-amyloid) in the brain induced by a physiological imbalance between the production and elimination of B-amyloid by B- and Y-secretases; this accumulation is followed by the development of neuropil threads, dystrophic neurites, associated astrogliosis, and microglial activation. The formation of these pathological plaques and the subsequent inflammation-mediated neurodegeneration are thought to inhibit neurotransmission [11].

However, the pathogenesis of AD is also influenced by genetic factors, and they are thought to trigger around 70% of all cases [3, 13]. Autosomal dominant disorders could also trigger some cases of AD, and the gene which produces amyloid precursor protein (APP) or the presenilins (PS1 or PS2) also produce a protein which harms vulnerable neurons, causing progressive dementia [14]. The APOE gene (apolipoprotein E) can occur in four variants: E1, E2, E3, and E4. The expression of the E4 form can increase the chance of promoting AD fourfold, with the odds ratio rising from 3 to 12 [15]. Under physiological conditions, APOE gene plays a fundamental role in cholesterol synthesis. In addition, the pathogenesis of AD has been associated with more than 20 different genes that play roles in the inflammatory process, endosomal vesicle recycling, and cholesterol metabolism [16].

Memantine was first approved by the FDA on 16 October 2003 and on 17 May 2002 in the European Union. It was first synthesized in Germany by Elly Lilly in 1968 as a potential antidiabetic drug; however, it was found to not be capable of lowering blood sugar level [17]. It was later discovered to have a potential influence on CNS activity, and was entered into clinical trials in 1986. Three years later, in 1989, it was confirmed to demonstrate uncompetitive blocking of NMDA receptors (N-methyl-D-aspartate) [16, 18, 19]. This receptor is one of the three classes of receptors (AMPA, Kainate, NMDA) which bind glutamate: a very important factor for physiological CNS functioning and one that acts as the main excitatory neurotransmitter in the mammalian brain. However, neurotransmitter activation is complex and requires the presence of both glycine and glutamate: the former remains at a constant level in the extracellular fluid, where it serves as a modulator, while glutamate is released from presynaptic stores in a constant manner, thus playing the role of neurotransmitter. Both are required to open integrated voltage-dependent calcium ion channels. Such depolarization removes the channel blockade provided by magnesium ions, which links the NMDA receptors to the electrical activity of the neuron and enables to connect neurotransmitter reproach to electrical state of neuron [20]. When NMDA transmission is sustained, it promotes various neuronal mechanisms, such as gene activation, and enhances learning and memory by ensuring synaptic plasticity. However, excess NMDA activation results in an increase in the levels of calcium ions, resulting in excitotoxicity [20, 21].

Memantine was initially believed to offer great promise: despite blocking NMDA, its administration did not entail specific NMDA antagonist side effects. This property has been attributed to the strong voltage dependency of this ligand [21]. Memantine is an uncompetitive weak antagonist of the NMDA receptor. It is bound to a slightly different region of the ion channel than magnesium ions, and with a slightly stronger bond [20, 21]. Despite this, the depolarization mechanism used to remove the blockade remains the same: the blockage becomes detached when depolarization is strong enough [21, 22]. Although a number of existing drugs, such as dizocilpine, ketamine, and phencyclidine, also noncompetitively inhibit NMDA receptors, they have not been registered for such indications. The problem is its kinetics: it binds easily to the PCP (phencyclidine) part of the NMDA receptors, but the connection is so strong that it does not permit physiological inhibition of the receptor by magnesium ions when receiving an impulse. This can result in psychosis, delusions, anxiety, and mainly negative symptoms due to excessive glutaminergic transmission [21, 23, 24].

Excessive glutaminergic activity plays a significant role in the pathogenesis of neurodegenerative disorders [22], due to an overabundance of information reaching the brain. Such a glut of information complicates the segregation and selection of correct information [22], as well as promoting necrosis of brain cells and increasing the level of apoptosis. On the other hand, strong blockage of NMDA receptors prevents the passage of impulses, and this is believed to lead to hallucinations or psychosis by the blocked impulses being replaced with fragments of different ones [20].

Controlled clinical trials have found memantine to be the first drug to alleviate the symptoms of dementia and to significantly slow down neurodegenerative processes. In addition, a number of clinical trials, some of them unfinished, have examined the potential use of memantine in the treatment of other neurological diseases. Memantine has a wide potential range of uses as a neuroprotective agent, and this range could be extended further. Today, the most promising additional use of memantine is in schizophrenia and depression.

## Schizophrenia

Schizophrenia is a psychotic disorder associated with damaged dopaminergic transmission in the brain [25]. It is characterized by a pathologically altered perception, unsettled connection with the external surroundings, and incorrect evaluation of external stimuli [25]. Psychotic patients demonstrate a significantly impaired ability to evaluate situations, state of mind, and thoughts, as well as social and family relationships [26]. Subjects can be unaware of the condition and may even refuse to accept it, which makes schizophrenia hard to treat. The condition impairs identity and most of the complex functions of human brain, resulting in great psychological and physical pain. Patients report that they feel they are “losing their mind” [25].

Although symptoms vary between patients, the core features are positive symptoms, such as delusions, hallucinations, and psychosis, which are attributable to excessive transmission in the mesolimbic dopamine system and negative symptoms, such as social withdrawal, and lack of spontaneous speech and motivation, that arise due to insufficient dopamine level in the mesocortical tract. Cognitive impairment is also present to greatly varying degrees. While the positive symptoms tend to arise temporarily as psychotic periods, the negative symptoms are more constant and chronic [27].

In many cases, this disease never resolves; however, a number of antipsychotic drugs have been discovered and they are helpful in alleviating the symptoms of schizophrenia and its commonly co-occurring conditions, such as depression, lack of motivation, or anxiety. The precise causes of schizophrenia remain unclear, but they are believed to be both environmental and genetic [25]. According to the neurodevelopmental hypothesis, the main paradigm for understanding environmental contributions, early neurodevelopment during pregnancy may play a key role in the development of the disease [28]. Factors such as maternal stress, infections, nutritional deficiencies during pregnancy, and birth or pregnancy complications may matter. In addition, the living environment of the growing child such as a city environment, socioeconomic factors, and the immigration of first and even second generation have also been associated with schizophrenia. Children born in late winter or early spring, or children with parents aged over 40 years or under 20 years, are also more likely to develop schizophrenia [28]. It is possible that these environmental agents can induce schizophrenia by affecting the expression of genes, but in this case the mechanism is more complex.

The first drugs developed to alleviate schizophrenia-associated symptoms are the neuroleptic or antipsychotic drugs such as chlorpromazine, thioridazine, or haloperidol. Those drugs differ slightly in terms of their activity or pharmacokinetics so they can be used in different kinds of schizophrenia or psychotic diseases. Nevertheless, they share a common mechanism of action. Neuroleptics act by inhibiting dopamine receptors, mainly D2 but also D1, D3, and D4, in accordance with the dopamine theory: the most commonly accepted theory in the epidemiology of schizophrenia [27]. However, these drugs also possess a number of side effects due to their inhibitory effects on other receptors, such as 5-HT-2, H1 M1, or adrenergic receptors [24]. The main problem associated with their use in treating schizophrenic patients is that they inhibit dopaminergic transmission throughout the brain.

Dopamine has a strong influence on human behavior, and large-scale blockage of dopamine receptors results in the development of side effects; therefore, although inhibiting dopamine receptors in mesolimbic tract may alleviate positive symptoms, this approach may also increase the strength of negative symptoms. It has been very difficult to obtain a drug which can intensify transmission in one part of the brain and decrease it in another. Although more selective drugs, such as clozapine or olanzapine, have been found to significantly reduce extrapyramidal symptoms, both positive and negative symptoms are inhibited by their action [24, 26, 29]. Despite the popularity of the dopamine theory, it does not fully account for the appearance of schizophrenia symptoms during excessive or decreased glutaminergic transmission. Hence, it has been proposed that schizophrenia may be caused by improper glutaminergic transmission in parts of the brain connected with the disease; this is known as the glutaminergic hypothesis [21, 27, 29–31]. This hypothesis is supported by evidence derived from clinical patients with established psychosis or hallucinations, as well as negative and other symptoms of schizophrenia, who were administered strong NMDA antagonists such as ketamine or dizocilpine. In addition, the NMDA receptors and glutaminergic tracts playing a role in schizophrenia can also be influenced on the genetic level [21, 28]. Furthermore, subcutaneous injections of NMDA antagonists caused neurodegenerative changes in rat brain cortex, similar to those occurring in the schizophrenic brain [21, 32].

Hence, memantine, an NMDA blocker and a strongly voltage-dependent compound which does not impair the physiological role of NMDA receptors, has been examined as a potential treatment for schizophrenia. Studies show it may have potential in treating both positive and negative symptoms of schizophrenia [30]. When administered as an add-on to antipsychotics, memantine shows significant alleviation of positive and negative symptoms recorded using the PANSS (Positive and Negative Symptom Scale) or Calgary Depression Scale of Schizophrenia (CDSS) tools [30]. In addition, double-blinded controlled placebo trials have shown a significant reduction of positive symptoms in patients suffering from schizophrenia compared with placebo without any serious side effects. The changes observed in the psychiatric scales above were not influenced by environment or the length of the trial [2]; however, it is important to emphasize that memantine was not used as monotherapy in those studies, but as an adjuvant to traditional antipsychotic therapy [21, 30, 32].

One study has shown that memantine may not only provide relief of the symptoms but could also modify the disease direction. Interestingly, significant differences between results have also been found between men and women [32]. These differences may come from physiological differences between males and females such as level of sex hormones, neurodevelopment, and also psychosocial factors. It is also claimed that females are more sensitive to neurotoxicity caused by glutamate [32]; however, it is important to emphasize that this trend is not shared by all antipsychotic drugs. It is worth adding that no significant difference in side effects was observed between memantine and placebo [31, 32], and that the side effects of memantine vary between people suffering from schizophrenia and those with Alzheimer’s disease, which may be related to differences in the psychological state [32]. Any lack of differences may be caused by the relatively short duration of the trial [32].

Memantine treatment has been found to alleviate not only the positive symptoms of schizophrenia, but also the negative symptoms; this is a promising result because the negative symptoms are the main reason for withdrawal from society and the lack of motivation which worsens overall clinical state of the patient [29]. One studied case found that adding memantine to risperidone therapy significantly decreased the degree of negative symptoms [26]. One case study found a patient to report improvement after 6 weeks on a regimen of 10 mg/day memantine. He was rated 96 on the SANS scale (Scale for the Assessment of Negative Symptoms), 3 in SAPS (Scale for the Assessment of Positive Symptoms), 3 in MMSE (Mini-Mental State Examination), and 2 in CDSS (Calgary Depression for Schizophrenia Scale). Following a dose increase to 20 mg/day, the typical dose for Alzheimer’s disease, he was rated SANS 76, SAPS 1, MMSE 26, and CDSS 1. Two months later, SANS had dropped to 70 and MMSE was 27, with SAPS and CDSS remaining the same [26]. No additional side effects were observed. The increased level of glutamate release in schizophrenia may be caused by NMDA receptor hypoactivity. For this reason, reversing this by NMDA receptor antagonist could represent a promising strategy to treating schizophrenia symptoms [33].

NMDA activity can also influence the course of schizophrenia by modulating the release of glutamate following the binding of serotonin or dopamine to 5HT_2a_ receptors [29, 34]. Atypical antipsychotics such as risperidone, found to alleviate positive symptoms almost completely in one studied case, can reduce hyperactivity of glutamate by binding to 5HT2a, and reduce dopamine release in the mesolimbic tract, and the combination of memantine and an atypical antipsychotic such as risperidone may prove an effective therapeutic option [29]. However, before any trial, it has been proposed that patients should be stabilized in terms of positive, extrapyramidal, and depression symptoms to ensure that any improvement can be attributed to pharmacotherapy [29].

Memantine also inhibits 5-HT-3 receptors, and it has been suggested that 5-HT-3 antagonists may improve negative symptoms [19, 34]. It may have a positive effect on negative, positive, and psychopathology functions but it pries results of the other trials [19]. Memantine was also found to offer promise in the treatment of catatonic schizophrenia: a case study found a patient to demonstrate significant improvement after memantine treatment, speaking more slowly and developing independence in daily routines such as shaving or taking a shower. This condition improved further with the discontinuation of other CNS drugs such as fluoxetine or donepezil, and greater improvement was observed at higher doses of memantine. This state deteriorated rapidly following discontinuation, but improved again after dose restoration. After 40 days of recovery, the patient was able to answer questions immediately and easily during normal conversation. Although no additional side effects were observed in this case, studies have found that memantine use may be associated with adverse reactions such as psychosis and seizures [35].

MRI studies of patients with schizophrenia have found brain activity to increase after adding memantine to antipsychotic therapy [31]. In contrast, other studies have found the addition of memantine did not significantly increase all positive or depressive symptoms; however, in these cases, the drug was discontinued or produced side effects such as dizziness, headache, constipation, or nausea [36]. Adding memantine to therapy in a group of patients taking risperidone showed less heterogeneity in terms of negative symptoms than in patients on other antipsychotics; this observation confirms the results of earlier studies. It was also found that younger patients displayed greater alleviation of symptoms [37]. Elsewhere, no significant difference was found between patients with schizophrenia on combined atypical antipsychotic plus memantine therapy and placebo with regard to outcome, and although memantine increases cognitive functions in patients with dementia, it does not appear to improve cognitive functions in healthy patients [36]. Although the study population may have been irregular, this does not exclude that memantine may be helpful in treating patients with more severe psychopathological disorders. Population included in the study was considerably younger and severity of symptoms was average. The study included a wide range of antipsychotics, and little is known about the interactions between memantine and other antipsychotic drugs [36].

The findings obtained by clinical trials vary slightly. Some cases show significant improvement in clinical state, after various periods of time. It shows potency in memantine in treating schizophrenia. These differences may be attributable to the presence of excessive or insufficient glutaminergic transmission [31]. Unfortunately, our understanding of the epidemiology of schizophrenia remains incomplete as the disease may vary considerably between different patients and many different types exist. Hence, a range of antipsychotics or antidepressant drugs is required to maintain a stable psychological condition, and this variation can significantly affect the results of a trial. Nevertheless, larger blinded, controlled, randomized trials and more focused research on schizophrenia epidemiology are needed to provide more targeted trials [31].

## Depression

Since the first published record of depressive disorders in psychiatric reviews in 1880, the prevalence of major depressive disorder has continued to grow [38].

A synthesized concept of mood disorders was published in 1899 by Kraepelin [39]. This was followed in 1934 by a manuscript describing depressive state as an unpleasant affect without organic changes in the brain or any schizophrenic disorder; these observations indicate that the affective changes appear primary, not secondary to other symptoms [40].

Depression affects 15–20% of the population and is a significant cause of morbidity worldwide due to suicide. It is associated with mood alteration, psychosocial impairment, and various neurochemical changes, and is always connected with diminished quality of life [38]. In some cases, it is also associated with cognitive impairment. Stress is also described as a factor which can affect the chance of developing depression [41, 42].

The treatment of major depressive disorder is quite complex because it is not a homogeneous state with different symptoms than may occur rarely; therefore, two opposite states can be the same symptom but it mainly concerns its intensity. Despite the heterogeneous character of the disease, some guidelines and schemes have been obtained to aid classification [43]. As each case is potentially unique, it is important to consider personal criteria such as tolerability when planning pharmacotherapy. Therapy should be adjusted to the type of the disorder and its dimension: for example, mood—sad, irritable, low, suicidal; activity or sleep—insomnia or hypersomnia [44].

Different types of depression exist, such as poststroke depression, postpartum depression, and posttraumatic depression, among others. [45–47]. The primary mechanism of depression is accounted for by the monoamine hypothesis, which claims that this state is the effect of insufficient level of noradrenaline and/or serotonin in the brain [48, 49]. Although today this theory is regarded as an oversimplification of depression, it nevertheless led to the discovery of drugs such as SSRI (selective serotonin reuptake inhibitors), SNRI (selective noradrenaline reuptake inhibitors), or monoamine oxidase inhibitors, which are in use today [50]. However, despite the fact that these drugs are still in use and they demonstrate positive clinical effects, some studies indicate they are not always successful [51]: other studies report poor tolerance of their adverse effects, and quite long and low remission rate [52, 53]. Further investigations are clearly needed to obtain more repeatable results and reliable therapies. With this in mind, as well as the potential of NMDA antagonists in treating psychiatric diseases such as schizophrenic depressive symptoms, memantine could represent a possible candidate for testing.

Some studies indicate that ketamine could be a promising agent in treating symptoms of major depressive disorder; however, this drug is not suitable for longer usage because of its addiction potential and strong psychomimetic character. No such properties have been observed for memantine; in addition, unlike the others, it can be administered orally [54].

Various clinical studies have examined the effects of NMDA antagonists. In one patient with very severe depressive symptoms, an infusion of ketamine was found to be helpful. His condition was initially poor: he had attempted suicide 10 times over the course of the previous 2 months and was resistant to various pharmacotherapy. Following treatment, the depressive symptoms were found to have been alleviated, as indicated by the Beck Depression Inventory (BDI) and 17-item Hamilton Depression Rating Scale (HDRS); however, the effect was transient and the symptoms returned after a short time. As multiple infusion was not recommended due to the addictive properties of ketamine, the regimen was supplemented with memantine. BDI and HDRS testing found that the symptoms had receded and the patient was discharged after 13 weeks [55]. These findings confirm available scientific data where memantine has been proven to be a good adjunctive antidepressant therapy [56]. Memantine has also been found to shorten the lag time between antidepressant administration and clinical effects, and to improve early therapy response [57]. The relationship between depression and the NMDA tract is further reinforced by the fact that people with MDD (major depressive disorder) also demonstrate higher glutamate levels in the brain and blood. Memantine has also been found to be effective in treating depression comorbid with alcohol abuse; however, escitalopram (SSRI) was much more effective [58]. Frequent alcohol consumption blocks NMDA receptors, thus increasing the regulation of these receptors, and contributing to alcohol tolerance [59].

The complexity of the role played by NMDA receptors in mood disorders confirming clinical studies implementing glutamate neurotransmission in mood disorders results in a wide range of potential areas of study [59]. Memantine also positively affects BDNF (brain-derived neurotrophic factor) production suggesting that glutamate plays an important role in the pathology and etiology of MDD [60–62]. It has also demonstrated synergistic effects when administered with different drugs: a 5-mg dose had an effect in Porsolt’s preclinical test. When it was administered alongside with sertraline, the effect was visible at any given dose. Biochemical tests of BDNF levels also showed the same tendency [62]. In the case of drug resistance, the dose was increased from the standard 20 to 40 mg with effective results; however, study group was quite low [63]. Elderly patients with dementia also demonstrated significant improvement in depressive symptoms when memantine was added to escitalopram treatment [64, 65]. In addition, the addition of memantine to imipramine in preclinical forced swimming test showed promising results at all doses [66]. In contrast, a meta-analysis of trials found memantine to not significantly reduce depressive symptoms in any case [67]. Such variation may be attributed to variations in the makeup of the population or trial exclusion criteria or to various physical issues, taking different drugs, or the reason of their depression [68].

The treatment of depression is complicated by its wide variety of etiologies. Depressive disorders may be triggered by chronic inflammation in brain associated with chronically increased levels of cytokines or other inflammatory mediators [69]. Such elevation induces a chain of endocrine reactions leading to neurotoxicity [70]. As neuroplasticity plays a significant role in the onset of depression, less neuroplasticity is associated with greater visibility of symptoms [71]. One approved SSRI, fluoxetine, increases neural plasticity and improves the dysfunctional glutamatergic neurotransmission visible in patients with MDD [72, 73].

A range of clinical trials is underway to clarify the potential of NMDA antagonists such as memantine in treating depression. Despite the fact that ketamine is known to alleviate depressive symptoms, it is not completely clear whether the glutamate tract plays the only role. More reliable data can be obtained with the use of larger studies employing validated exclusion criteria or more homogenous patients. [74, 75]

## Summary

Memantine is effective in treating some nervous system disorders and appears to be well tolerated by patients. The combination of memantine and various atypical antipsychotics also appears to offer great potential. Memantine also helps alleviate symptoms of dementia and significantly slows neurodegenerative processes. However, as psychiatric cases are so heterogeneous and require the individual treatment of almost every patient, further clinical studies are still needed. Nevertheless, the results of ongoing trials are very promising, with the majority yielding positive outcomes.
